# Human health risk of nitrate in groundwater of Tehran–Karaj plain, Iran

**DOI:** 10.1038/s41598-024-58290-6

**Published:** 2024-04-03

**Authors:** Maedeh Alizadeh, Roohollah Noori, Babak Omidvar, Ahmad Nohegar, Severin Pistre

**Affiliations:** 1 Graduate Faculty of Environment, University of Tehran, Tehran, 1417853111 Iran; 2 Faculty of Governance, University of Tehran, Tehran, 1439814151 Iran; 3 HydroSciences Montpellier, University of Montpellier, CNRS, IRD, 34090 Montpellier, France

**Keywords:** Environmental sciences, Hydrology

## Abstract

Groundwater pollution by nitrate has is a major concern in the Tehran–Karaj aquifer, Iran, where the wells provide up to 80% of the water supply for a population of more than 18 million—yet detailed human health risks associated with nitrate are unknown due to the lack of accessible data to adequately cover the aquifer in both place and time. Here, using a rich dataset measured annually in more than 75 wells, we mapped the non-carcinogenic risk of nitrate in the aquifer between 2007 and 2018, a window with the most extensive anthropogenic activities in this region. Nitrate concentration varied from ~ 6 to ~ 150 mg/L, around three times greater than the standard level for drinking use, i.e. 50 mg/L. Samples with a non-carcinogenic risk of nitrate, which mainly located in the eastern parts of the study region, threatened children’s health, the most vulnerable age group, in almost all of the years during the study period. Our findings revealed that the number of samples with a positive risk of nitrate for adults decreased in the aquifer from 2007 (17 wells) to 2018 (6 wells). Although we hypothesized that unsustainable agricultural practices, the growing population, and increased industrial activities could have increased the nitrate level in the Tehran-Karaj aquifer, improved sanitation infrastructures helped to prevent the intensification of nitrate pollution in the aquifer during the study period. Our compilation of annually mapped non-carcinogenic risks of nitrate is beneficial for local authorities to understand the high-risk zones in the aquifer and for the formulation of policy actions to protect the human health of people who use groundwater for drinking and other purposes in this densely populated region.

## Introduction

Clean-groundwater, as a key component of the hydrological cycle, sustains life on our planet^1–3^. According to the 2018 edition of the World Water Development Report (WWDR)^4^ of the United Nations, safe access to clean-groundwater is at risk globally, and the situation will aggravate by the introduction of more pollution loads to the environment in the coming decades. Our understanding of the groundwater quality is less than that of the groundwater quantity^5,6^, although both are equally important in the context of sustainable management of water resources^7^. This issue has been also highlighted in the 2018 edition of the WWDR^4^, where the associated sections with the state of groundwater quality have been poorly supported^4^. Therefore, further investigations are needed to appropriately understand the state of groundwater quality, especially in developing nations because approximately 80% of the deaths and diseases in these countries are associated with water pollution^8^.

Among the various chemical compounds in groundwater, which may affect human health, nitrate is the most common^9–12^. High nitrate levels in drinking water, particularly in groundwater, have been frequently caused by increased anthropogenic activities such as agriculture and manufacturing nitrogen inputs^13–17^. Many countries throughout the globe have high nitrate levels in their drinking water, including Iran^18,19^, India^20^, and China^21^. As a result, nitrate poisoning is becoming more widespread in groundwater basins under farmlands with well-drained soils and oxic geochemical conditions^22,23^. Although methemoglobinemia (blue-baby syndrome) is the most immediate life-threatening result of nitrate exposure from contaminated water (particularly for babies), additional significant effects have been identified, including 15 forms of cancer and two types of birth abnormalities^19,24–28^. The most common way that nitrate enters the body is through drinking water. The human body’s internal processes convert the ingested nitrate into nitrite, which may oxidize Fe^2+^ present in blood cells into Fe^3+^, forming methemoglobin, a molecule unable to carry oxygen^25,29^. The health of the consumer is at risk because methemoglobin production causes anoxia in the body’s organs^30,31^. Additionally, nitros-amines/amides, which have both carcinogenic and non-carcinogenic impacts, may be produced when nitrite bonds with amines and amides^32–35^. Besides, a recently conducted research work has reported that nitrate can release some radioactive elements such as uranium into the groundwater^36^. All in all, the World Health Organization (WHO)^37^ and the Institute of Standards and Industrial Research of Iran (ISIRI)^38^, have set the upper limit of nitrate concentration at 50 mg/L (or about 11 mg/L in terms of nitrogen) to safeguard the human health against the harmful impacts of this contaminant in drinking water.

The challenges related to the clean-groundwater supply associated with nitrate are more severe in the densely populated regions located in developing countries such as Iran, particularly in the Tehran–Karaj aquifer^39–43^. Tehran–Karaj plain has struggled with issues like rapid urbanization, rising population, increased agricultural and industrial activities, and unbalanced use of fertilizers, which led to elevated nitrate in groundwater^44^. Access to the clean-groundwater associated with nitrate is now the crucial concern of the Tehran–Karaj water authorities, where the groundwater supplies up to 80% of the water demands during dry seasons, for approximately 20% of the country’s population who resident in the plain. Notwithstanding the importance of the Tehran–Karaj aquifer in supporting life in the densely populated region, detailed human health risks associated with nitrate are not clear due to the lack of accessible data to adequately cover both place and time in the plain. For example, Noori et al.^15^ investigated both carcinogenic and non-carcinogenic health risks of nitrate in 100 tap water samples taken from Tehran’s city in 2018. Kalteh et al.^44^ assessed non-carcinogenic health risk of nitrate in 148 tap water samples taken from six districts of Tehran’s city in 2022. In another conducted study, Badihi et al.^45^ used 66 drinking water samples taken from southwest region of Tehran in 2020 to assess their non-carcinogenic health risks of nitrate for end-users. However, a few studies have investigated the human health risk of nitrate in the groundwater wells using sporadic data that covered only some parts of the aquifer in a short window^15,44,45^. In addition, the plain has exposed to an elevated production of pollution loads due to expanded urbanization and industrial-agricultural development, which could further rich the aquifer with nitrate concentration during the study period. On the other hand, the improved sanitation infrastructures (e.g., construction of wastewater treatment plants and improved coverage of the sewage collection network) could conserve the aquifer from the introduced pollution loads. However, generalization of the results from the conducted studies to a period in that the plain has experienced extensive human-made activities (i.e., 2002 onwards^46^) is difficult. Here, using a rich dataset measured seasonally/annually in more than 75 wells, we aim to (i) evaluate the nitrate concentration in the aquifer to distinguish the wells with the elevated concentration above the permissible level for drinking water during the study period (2007–2018), (ii) map the spatial distribution of nitrate concentration and its associated non-carcinogenic risks from 2007 to 2018, a window with the most extensive anthropogenic activities in the plain^46^, and (iii) to understand whether the nitrate has raised or declined in the aquifer.

## Materials and methods

### Study area

**Figure 1:**
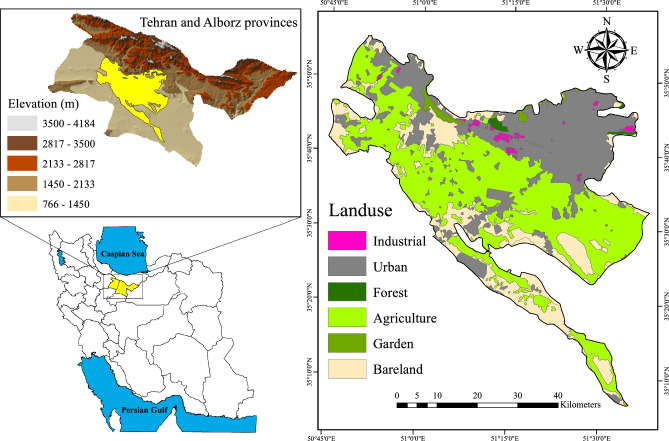
The study area of the Tehran–Karaj aquifer along with the land-use/cover map (This figure was prepared in ArcGIS, version 10.8).

The study area, the Tehran–Karaj aquifer, with an area of ~ 2570 km^2^ is located between 50° 45′ and 51° 37′ Eastern longitudes and between 35° 13′ and 35° 52′ Northern latitudes (Fig. 1). The presence of Alborz Mountain ranges in the region results in a variety of climate patterns. In terms of climatic classification, the north is semi-cold, while the weather gradually turns semi-arid as one moves toward the middle and south^47^. Tehran, the capital, and Karaj megacities are located in this plain, and groundwater supplies over half of the water needs for both cities^48^.

The Tehran–Karaj aquifer is a one-layer unconfined aquifer with a thickness that varies between 50 m in the north and 300 m in the center, and it decreases to 100 m in the southern plain. The aquifer transmissivity ranges from 200 to 2500 m^2^ per day^49,50^. According to observation well logs, geology maps, and geophysical study results, groundwater depth in the studied wells varied from a few meters (e.g., 5 m) to more than 250 m during the study period^49^. The Tehran–Karaj plain’s slope is more in the north than in the south. The majority of the area’s central and southern regions have low slopes and are nearly flat terrain^50^. Generally, water flows from the northern boundary, which is next to the Alborz mountains, to the southeastern part of this aquifer^51^. Precipitation (~ 250 mm/yr), surface water sources (e.g., Karaj river and Kan river), disposal of urban and industrial effluents through pit latrines, and irrigation return flows are the main source of recharge for this aquifer. However, these sources of recharge have declined, mainly due to frequent droughts and improved coverage of sewage collection network in the plain during two last decades^50,52–55^.

**Figure 2:**
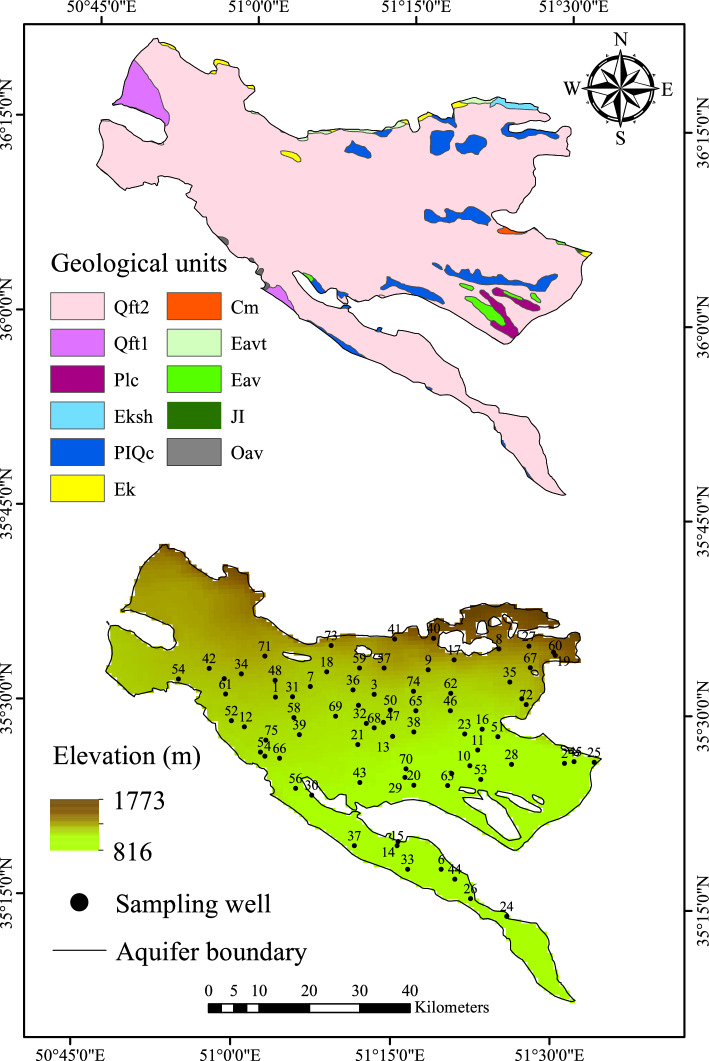
Geology map of the Tehran–Karaj aquifer and the map of sampling locations (groundwater wells) (This figure was prepared in ArcGIS, version 10.8). Qft2: Low-level piedment fan and vally terrace deposits; Qft1: High-level piedmont fan and vally terrace deposits; Plc: Polymictic conglomerate and sandstone; Eksh: Greenish black shale; PlQc: Fluvial/Piedmont conglomerate and sandstone; Ek: Well-bedded green tuff and tuffaceous shale; Cm: Dark-grey to black fossiliferous limestone; Eavt: Andesitic volcanic tuff; Eav: Andesitic volcanics; JI: Light-grey, thin-bedded to massive limestone; and Oav: Oligocene andesitic lava flows.

In term of geology, the geological structures of the studied area have been heavily impacted by the tectonic activity of the Alborz active zone, which has resulted in considerable rising folding and faulting with an east–west trend. The tectonic formations in the plain have been shaped by the formation of sedimentary rocks and new clastic deposits. Pleistocene clastic deposits make up the surface parts of the Tehran–Karaj plain. Low-level piedmont fan and vally terrace deposits dominantly cover the study area, followed by fluvial/piedmont conglomerates and sandstones. In the northwest, there are high-level piedmont fan and vally terrace deposits. Polymictic conglomerate and sandstones (formed during the Pliocene), greenish/black shale (formed during Middle Eocene), and dark-grey to black fossiliferous limestone (formed during Carboniferous) are mainly located in the eastern region of the plain. Well-bedded green tuff and tuffaceous shale (formed during the Eocene) can be observed in the north end (Fig. 2). In the northern hillsides of Alborz, the sediments are coarser in grain size compared to the finer sediments in the southern parts^48^.

As shown in the land-use/cover map extracted from the archive of Iran’s Ministry of Agriculture Jihad (Fig. 1), the study area is primarily made up of agricultural fields, with smaller amounts of urban and industrial zones. The concentration of the rural populace and agricultural territories is notably elevated in this specific region. In addition, a multitude of factories and industrial hubs such as Iran’s largest automobile manufacturers are mainly situated in the western part of the aquifer. Tehran and Alborz provinces also host several large chemical and petrochemical complexes in the south. Additionally, the area has a large number of irrigation and drinking wells that provide water needs for Tehran and Alborz provinces^11^. This region comprises areas that are impacted by agricultural pesticides and both organic and chemical nitrogen-rich fertilizers. The agricultural fields in the study region meet the majority of Tehran and Alborz’s food demands, including vegetables and citrus fruits^40^.

### Data

In this study, 1705 groundwater nitrate samples from 107 representatives (semi)deep wells taken from the Tehran–Karaj aquifer within the 12 years monitoring period (2007–2018) were used. This dataset was archived by the Iran’s Water Resources Management Company (IWRMC), a government organization responsible for water-related data as a subsidiary of Ministry of Energy. The IWRMC seasonally/annually measures nitrate concentration in this aquifer. This organization performs quality assurance procedure (e.g., procedure documentation, equipment calibration process, and staff training) according to (inter)national guidelines to verify the accuracy of measured data. The IWRMC also performs quality control (e.g., blanks, spikes, replicates, calibration standards, and performance audits) regularly to insure that the sampling analysis’s results are assured to be accurate within the allowable limits and referenced standards. However, the final results should satisfy a rate of nitrate recovery within ± 10%.

The number of nitrate samples in almost wells (> 95%) was taken more than once per year. Therefore, throughout our analysis, we considered the annual mean value of nitrate concentrations for individual wells. To satisfy the requirements for trend analysis (see section “Statistical methods”), we removed wells with less than ten years of data during the study period, which led to a reduction of 107 to 75 wells, as shown in Fig. 2. The selected 75 wells, which are used to supply drinking, industrial, and agriculture water, appropriately distributed across Tehran–Karaj aquifer. The raw data are included in a “Zenodo” repository, available through https://zenodo.org/deposit/8179225.

After each sampling campaign, water samples were acidified by sulfuric acid, H_2_SO_4_, (pH < 2), and filtered through a fine membrane filter with a diameter < 0.45 µm. Thereafter, water samples were stored in glass vessels, kept in a cool box (temperature = ~ 4 °C), and transferred to the laboratory certified by the Iran Department of Environment. To ensure quality assurance, nitrate analysis was carried out in agreement with the Standard Methods’ guidelines specified for the examination of (waste) water^56^. Nitrate in the collected water samples was analyzed by both Hach DR/2800 and DR/5000™ UV–Vis Spectrophotometers with Hach method 10,020. The detection limit of Hach method 10,020 is 0.3 mg/L.

### Health risk assessment

It is beneficial to assess the health-related risk of chemicals in contaminated drinking waters to understand the likelihood of adverse health effects. Risk assessment is often the first step in safeguarding human safety and health^28,57,58^. In this study, we used the empirical model proposed by the U. S. Environmental Protection Agency (USEPA)^59^, to estimate nitrate’s non-carcinogenic risks in the groundwater resources of the Tehran–Karaj aquifer. Based on physiological and behavioral differences, the population was divided into three age groups as follows: children, teenagers, and adults. Then, the daily nitrate exposure via drinking water in these groups was calculated using Eq. (1)^60^.

1 $${\text{EDI}}= \frac{{{\text{C}}}_{\mathrm{f }}\times {{\text{C}}}_{{\text{d}}} }{{{\text{B}}}_{{\text{w}}}}$$

where, EDI stands for the estimation of daily nitrate consumption (mg/kg), C_f_ is the annual mean nitrate concentration in drinking water (mg/L) for each well, C_d_ is the average daily drinking water intake (liter), and B_w_ is the body weight (kg).

The non-carcinogenic impact of a single element can be expressed as the hazard quotient (HQ) using Eq. (2):

2 $${\text{HQ}}= \frac{{\text{EDI}}}{{\text{RFD}}}$$

where RFD stands for the reference dose (mg/kg d).

The RFD is 1.6 mg/kg.d for nitrate from the digestive tract^61^. A value of HQ < 1 indicates that no harmful effects of exposure are expected, whereas a value of HQ > 1 indicates that the non-carcinogenic risk exceeds the acceptable level^61,62^. Table 1 displays the formula parameter values for the various exposed groups.

**Table 1:** Values of parameters that are used for nitrate health risk assessment in this study^61,62^.

Group	C_f_ (mg/L)	C_d_ (L/d)	B_w_ (kg)	RFD (mg/kg d)
Children	**–**	0.85	15	1.6
Teenagers	**–**	2	50	1.6
Adults	**–**	2.5	78	1.6

### Statistical methods

In this study, we used Mann–Kendall (MK)^63^ and Theil-Sen estimator (SSE)^64^ methods to explore univariate trends in the health risks of nitrate exposed to groundwater samples for different age groups. All measured data were used, and no reconstruction technique was used to complete the sampling points with no data.

#### Trend detection

The MK test was employed for the detection of trends in data sets. In this regard, the null hypothesis was that the data were represented by a set of m randomly ordered independent variables and there is no trend in the data. This test is done using Eq. (3):

3 $$s = \mathop \sum \limits_{k = 1}^{m - 1} \mathop \sum \limits_{j = k + 1}^{m} {\text{sgn}}\left( {x_{j} - x_{k} } \right),{ }\quad {\text{where}},\quad {\text{sgn}}\left( x \right) = \left\{ {\begin{array}{*{20}l} 1 \hfill & { if \, x > 0} \hfill \\ {0 } \hfill & { if \, x = 0} \hfill \\ { - 1 } \hfill & { if \, x < 0} \hfill \\ \end{array} } \right.$$

where *m* is the number of data points, and the mean and variance of *S* are zero and unit, respectively. For *m* > 10, *S* is transformed into the standard normal variable *Z* through Eq. (4)^65^:

4 $$Z = \left\{ {\begin{array}{*{20}l} {\frac{S - 1}{\sigma }} \hfill & { if\, S > 0} \hfill \\ 0 \hfill & { if\, S = 0} \hfill \\ {\frac{S + 1}{\sigma } } \hfill & { if \, S < 0} \hfill \\ \end{array} } \right.$$

where *σ* is the standard deviation of *S*.

In Eq. (4), positive and negative values show increasing and decreasing trends detected by the MK test in the applied dataset, respectively.

#### The slope of trend calculation

The SSE is widely used to determine the slope of a trend in a historical dataset as it is not highly affected by missing data and outliers^66^. SSE calculations start with estimating the slope of *k* pairs of data using Eq. (5):

5 $$Q_{i} = \frac{{x_{j} - x_{k} }}{j - k},\quad i = 1,{ }2, \ldots ,k$$

where *Q* is the slope between data points measured at times *j* (${x}_{j}$) and *k* (${x}_{k}$) with *j* > *k*. The Sen’s slope is then computed as the median value of ${Q}_{i}$ using Eqs. (5) and (6) for odd and even pairs of data, respectively.

6 $$Q_{med} = Q_{{\left[ {\left( {k + 1} \right)/2} \right]}} \quad Q_{med} = 0.5\left( {Q_{{\left[ {k/2} \right]}} + Q_{{\left[ {\left( {k + 1} \right)/2} \right]}} } \right)Q_{{\left[ {\left( {k + 1} \right)/2} \right]}}$$

Thereafter, the *Q*_*med*_ is tested by a two-sided test at the 100(1 − α)% confidence interval and the true slope is calculated by the non-parametric test^67^. Here, the upper and lower confidence limits for Sen’s slope were calculated by considering the confidence interval equal to 0.1.

The MK and SSE methods were performed using MAKESENS 1.0, a user-friendly code developed Excel developed by the Finnish Meteorological Institute^68^, accessible via https://en.ilmatieteenlaitos.fi/makesens. More details about the SSE method are given in Partal and Kahya^67^.

#### Spatial distribution

The spatial distribution of nitrate content in the Tehran–Karaj aquifer was examined using the ArcGIS 10.8 program and an inverse distance weighting (IDW) interpolation approach. The IDW is a method that determines interpolation based on the mean weight of each variable and the distance between locations. This method is more effective in groundwater quality studies than other relevant techniques (e.g., Kriging) because it highlights the high extreme values that can expose human health to contaminants^69^.

## Results

### Nitrate concentration

The statistical summary of nitrate concentration in 75 wells during the studied period (2007–2018) is given in Table 2. The results revealed that the highest level of nitrate was observed in 2010 (i.e., 148.8 mg/L). The fluctuation of nitrate concentration showed that the maximum amount of this ion increased from the beginning year of the study period (i.e., 2007) to 2010, and it then declined by 2013. Afterwards, the maximum concentration of nitrate increased by 2015. Subsequently, the nitrate decreased by the end of study period (i.e., 2018) (Table 2). The results also showed that the nitrate concentrations were higher than 50 mg/L, the standard level determined for drinking consumption by the WHO^37^, in around 24% in 2007, 19% in 2008, 17% in 2009, 18% in 2010, 17% in 2011, 10% in 2012, 19% in 2013, 21% in 2014, 17% in both 2015 and 2016, 16% in 2017, and 10% in 2018.

**Table 2:** Statistical characteristics of nitrate concentration in the studied wells in the Tehran–Karaj aquifer during the study period (2007–2018). St.D stands for the standard deviation.

Sampling year	Mean (mg/L)	St.D (mg/L)	Max (mg/L)	Median (mg/L)
2007	34.1	26.1	102.3	24.8
2008	33.5	26.1	120.9	24.8
2009	36.1	28.2	136.4	27.9
2010	36.2	30.3	148.8	24.8
2011	32.7	22.6	117.8	26.3
2012	28.3	17.2	89.9	24.8
2013	29.3	16.9	68.2	21.7
2014	36.9	24.6	117.8	27.9
2015	32.1	20.1	130.2	27.9
2016	34.9	19.8	120.9	31.0
2017	34.7	18.4	111.6	31.0
2018	34.0	13.7	68.2	34.1

**Figure 3:**
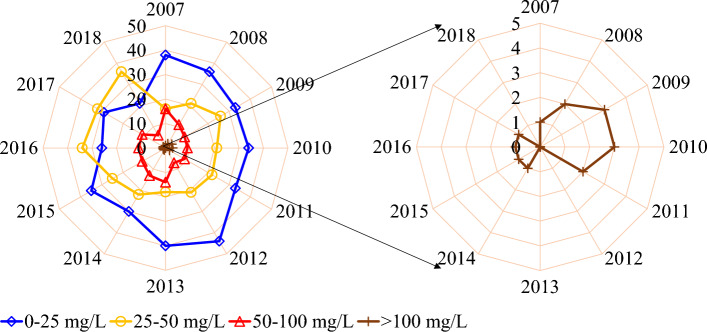
The number of wells that fall into different ranges of nitrate concentration during the study period (2007–2018) (This figure was prepared in Microsoft Excel).

In 2012, 65 groundwater wells had safe nitrate levels below 50 mg/L, which decreased to 52 wells by 2014. The highest number of wells with nitrate concentrations between 50 and 100 mg/L was recorded in 2007 (16 wells), decreasing to a minimum of 6 wells in 2018. Although all wells had nitrate concentrations below 100 mg/L in 2012, 2013, and 2018, three out of 75 wells showed concentrations higher than 100 mg/L in 2009 and 2010. There was also a decrease in the number of wells with nitrate concentrations below 25 mg/L during the study period (except in 2012 and 2015) (Fig. 3).

**Figure 4:**
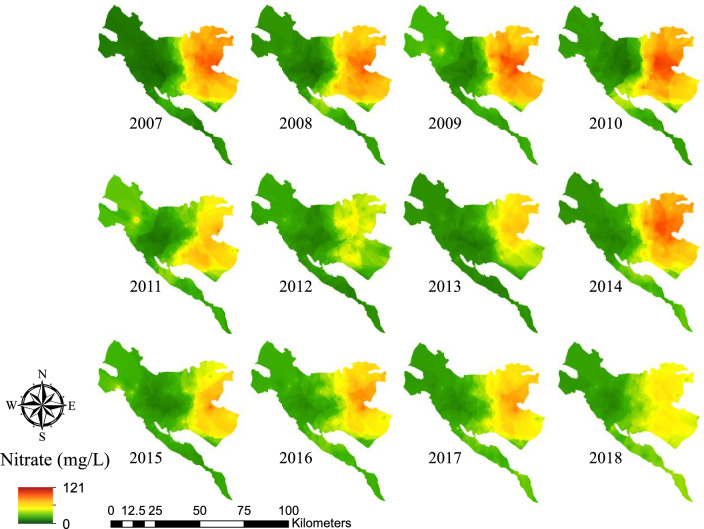
Spatial distribution of nitrate in Tehran–Karaj aquifer (2007–2018) (This figure was prepared in ArcGIS, version 10.8).

Figure 4 depicts the spatial distribution of nitrate in the study area from 2007 to 2018. This figure shows that the eastern region of the aquifer has experienced larger concentrations of nitrate, and hence; it may face a high human health risk. The highest nitrate concentration in the Tehran–Karaj aquifer in the study period (i.e., 148.8 mg/L, Table 2) was also recorded in the east of the aquifer in 2010. This figure also suggests that the nitrate concentrations in the eastern region of the aquifer have decreased from 2007 to 2018.

### Health risk of nitrate in the aquifer

**Figure 5:**
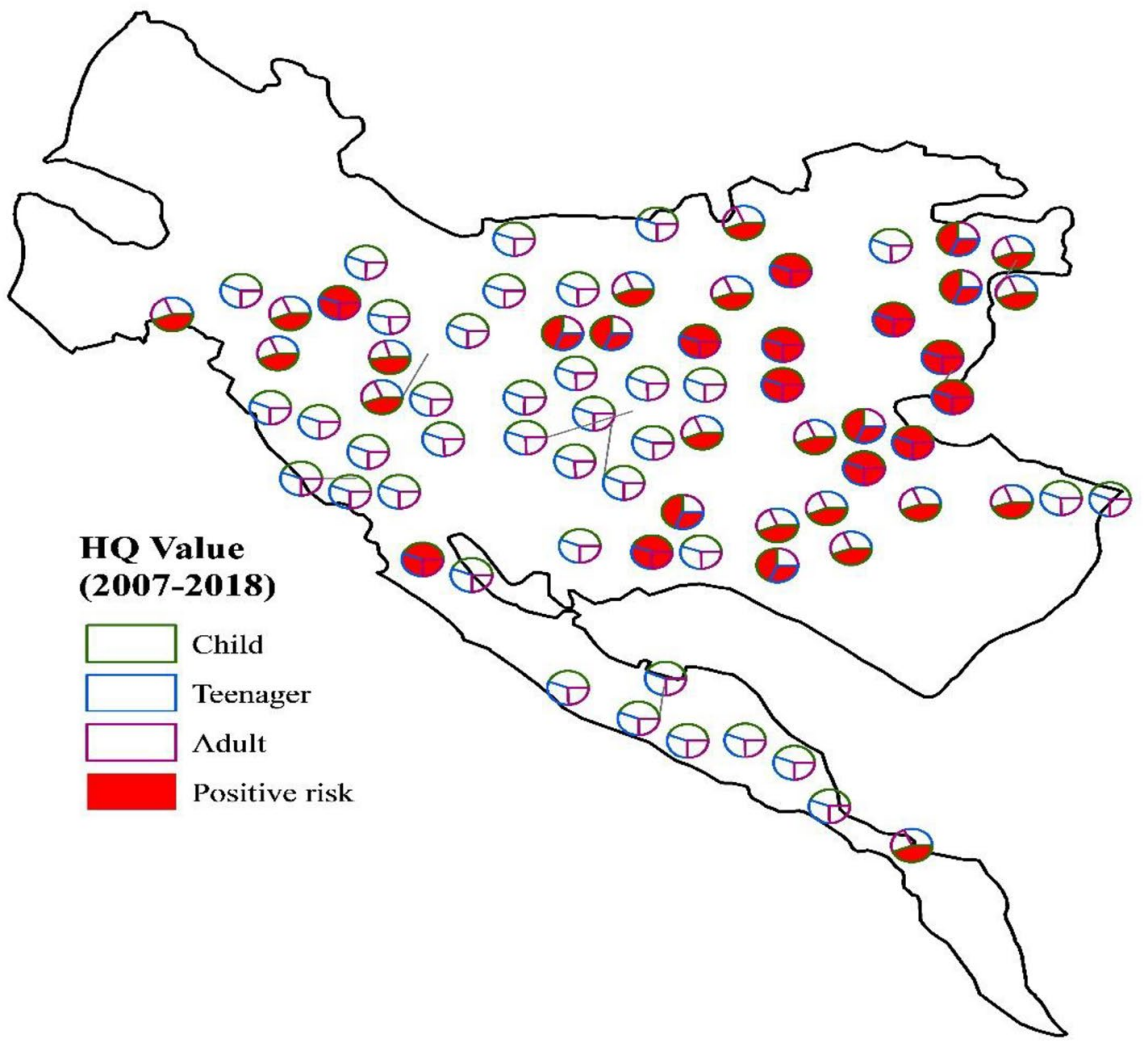
Averaged spatial distribution of the hazard quotient (HQ) associated with nitrate measured in the Tehran–Karaj aquifer for different age groups (i.e., children, teenagers, and adults) during the study period (2007–2018) (This figure was prepared in ArcGIS, version 10.8).

In this study, the non-carcinogenic health risks of nitrate concentration in Tehran–Karaj groundwater wells were investigated. In the first step, the estimated daily intake (EDI, mg/kg/d) and hazard quotient (HQ) values of nitrate for different age groups (i.e., children, teenagers, and adults) were calculated by using Eqs. (1) and (2). The mean and maximum HQ values for various population groups during each year are provided in Table 3, suggesting the HQ levels increased in the following order: children > teenagers > adults. Children are the most vulnerable groups in the exposed population investigated, which was negatively affected by nitrate in drinking water in almost all years. However, the HQ value averaged from the samples for children in 2012 was 0.96, a value close to the threshold level suggested by the USEPA^59^ for the non-carcinogenic risk of nitrate in drinking water. Further investigations revealed that samples with the non-carcinogenic risk of nitrate for children, teenagers, and adults (HQ > 1) were mainly located in the eastern part of the study region (Fig. 5), where the maximum annual concentrations of nitrate can be observed, as shown in Fig. 4, from 2007 to 2018.

**Table 3:** Statistically analyzed water estimated daily intake (EDI) and hazard quotient (HQ) of nitrate concentration for children, teenagers, and adults during the study period (2007–2018).

Year	Statistical index	EDI			HQ		
		Children	Teenager	Adult	Children	Teenager	Adult
2007	Mean	1.8	1.3	1.0	1.1	0.8	0.6
	Max	5.8	4.1	3.3	3.6	2.6	2.1
2008	Mean	1.8	1.3	1.0	1.1	0.8	0.6
	Max	6.9	4.8	3.9	4.3	3.0	2.4
2009	Mean	1.9	1.4	1.1	1.2	0.9	0.7
	Max	7.7	5.5	4.4	4.8	3.4	2.7
2010	Mean	1.8	1.3	1.0	1.1	0.8	0.6
	Max	8.4	6.0	4.8	5.3	3.7	3.0
2011	Mean	1.6	1.2	0.9	1.0	0.7	0.6
	Max	6.7	4.7	3.8	4.2	3.0	2.4
2012	Mean	1.5	1.1	0.9	1.0	0.7	0.5
	Max	5.1	3.6	2.9	3.2	2.3	1.8
2013	Mean	1.6	1.1	0.9	1.0	0.7	0.6
	Max	3.9	2.7	2.2	2.4	1.7	1.4
2014	Mean	1.8	1.3	1.0	1.2	0.8	0.7
	Max	6.7	4.7	3.8	4.2	3.0	2.4
2015	Mean	1.7	1.2	1.0	1.1	0.8	0.6
	Max	7.4	5.2	4.2	4.6	3.3	2.6
2016	Mean	1.9	1.3	1.1	1.2	0.8	0.7
	Max	6.9	4.8	3.9	4.3	3.0	2.4
2017	Mean	1.9	1.3	1.1	1.2	0.8	0.7
	Max	6.3	4.5	3.6	4.0	2.8	2.2
2018	Mean	1.6	1.1	0.9	1.0	0.7	0.6
	Max	3.9	2.7	2.2	2.4	1.7	1.4

**Figure 6:**
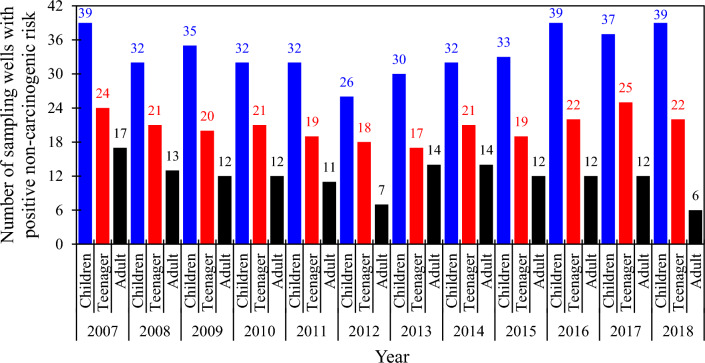
The number of sampling locations exposed to the non-carcinogenic risk of nitrate in the Tehran–Karaj aquifer (This figure was prepared in Microsoft Excel).

The HQ values averaged over the study region reveal that the number of samples with non-carcinogenic risk of nitrate (HQ > 1) fluctuates from 2007 to 2018. For example, our results show that around half of the groundwater samples are not safe for children’s drinking water-use in the last years of the study period (i.e., 2016, 2017, and 2018). The maximum number of safe wells associated with non-carcinogenic risk of nitrate for adults is observed at the end of the study period in 2018. Detailed information about the number of wells with non-carcinogenic risk of nitrate for different age groups during the study period is shown in Fig. 6.

### Evolution of nitrate health risk for different age groups

Table 4 shows the temporal trend of HQ for three age groups (i.e., children, teenagers and, adults) in 75 selected wells in the Tehran–Karaj aquifer. The results show no significant change of HQ in the most studied wells for age groups during the study period. However, around 23 and 8 wells have experienced an increasing and decreasing trend of HQ, respectively, for different age groups.

**Table 4:** Magnitude (slope) and direction (−/+) of trends for the hazard quotient (HQ) associated with nitrate measured in each studied well located in the Tehran–Karaj aquifer for different age groups (i.e., children, teenagers, and adults) during the study period (2007–2018).

Well No	Children	Teenagers	Adults	Well No	Children	Teenagers	Adults
1	**− 0.146**	**− 0.103**	**− 0.083**	39	**0.03**	**0.02**	**0.02**
2	− 0.009	− 0.007	− 0.005	40	**− 0.11**	**− 0.08**	**− 0.06**
3	0.000	0.000	0.000	41	0.00	0.00	0.00
4	0.000	0.000	0.000	42	− 0.02	− 0.01	− 0.01
5	0.018	0.013	0.010	43	0.00	0.00	0.00
6	**0.041**	**0.029**	**0.023**	44	**0.04**	**0.03**	**0.02**
7	**0.032**	**0.023**	**0.018**	45	− 0.02	− 0.02	− 0.01
8	0.000	0.000	0.000	46	− 0.03	− 0.02	− 0.02
9	0.031	0.022	0.018	47	**0.05**	**0.04**	**0.03**
10	− 0.026	− 0.018	− 0.015	48	**0.04**	**0.03**	**0.02**
11	**− 0.110**	**− 0.077**	**− 0.062**	49	− 0.05	− 0.04	− 0.03
12	0.037	0.026	**0.031**	50	0.02	0.01	0.01
13	0.014	0.010	0.008	51	− 0.01	− 0.01	− 0.01
14	0.000	0.000	0.000	52	**0.05**	**0.04**	**0.03**
15	**0.066**	**0.047**	**0.037**	53	− 0.05	− 0.04	− 0.03
16	0.000	0.000	0.000	54	0.00	0.00	0.00
17	0.049	0.034	0.028	55	0.02	0.01	0.01
18	**0.029**	**0.020**	**0.016**	56	0.00	0.00	0.00
19	0.027	0.019	0.016	57	0.01	0.01	0.01
20	0.000	0.000	0.000	58	**0.03**	**0.02**	**0.02**
21	0.000	0.000	0.000	59	0.00	0.00	0.00
22	**0.044**	**0.031**	**0.025**	60	0.00	0.00	0.00
23	− 0.037	− 0.026	− 0.021	61	0.06	0.04	0.03
24	**0.055**	**0.039**	**0.031**	62	**− 0.15**	**− 0.11**	**− 0.09**
25	0.00	0.00	0.00	63	**0.05**	**0.03**	**0.03**
26	**0.05**	**0.04**	**0.03**	64	**− 0.11**	**− 0.08**	**− 0.06**
27	0.02	0.01	0.01	65	− 0.01	− 0.01	0.00
28	− 0.05	− 0.04	− 0.03	66	**0.05**	**0.04**	**0.03**
29	**− 0.14**	**− 0.10**	**− 0.08**	67	0.00	0.00	0.00
30	**0.04**	**0.03**	**0.02**	68	**0.07**	**0.05**	**0.04**
31	− 0.01	− 0.01	− 0.01	69	**0.03**	**0.02**	**0.02**
32	**0.11**	**0.08**	**0.06**	70	**− 0.08**	**− 0.06**	**− 0.05**
33	0.01	0.01	0.01	71	**0.05**	**0.04**	**0.03**
34	0.02	0.00	0.00	72	− 0.08	− 0.06	− 0.05
35	− 0.08	− 0.06	− 0.05	73	**0.07**	**0.05**	**0.04**
36	**0.11**	**0.08**	**0.06**	74	**− 0.16**	**− 0.11**	**− 0.09**
37	0.00	0.00	0.00	75	**0.04**	**0.03**	**0.02**
38	− 0.03	− 0.02	− 0.02				

Statistically significant trends have been shown by a bold font.

## Discussion

Tehran–Karaj aquifer is an important source of water for around 20% of Iran’s population who resides in Tehran and Alborz provinces^15^. However, this aquifer is prone to contamination by nitrate due to extensive urbanization, unsustainable agricultural practices, and unregulated inputs of raw sanitary and industrial effluents^15,41,52^. Here, we evaluated nitrate concentration in the Tehran–Karaj aquifer and found that some wells, which mainly located in the eastern part of the study region, were unsafe with respect to the non-carcinogenic risk of nitrate for different age groups. It should be noted that no local information about the C_d_, B_w_, and RFD was available in our case study. Therefore, we had to use the corresponding reference values suggested by the USEPA, as given in Table 1, for estimation of the human health risks. It could introduce some degree of uncertainty in our presented results for Tehran–Karaj aquifer. However, these reference values have been widely used in the studies conducted around the world, where no local data were available to calculate the human health risk of nitrate in drinking water^70–72^.

Spatial distribution of nitrate in the Tehran–Karaj aquifer revealed that the groundwater resources in the eastern region were more polluted with nitrate compared to other regions (Fig. 4), where the aquifer is mainly covered by Tehran city in the east and northeast and the agricultural lands in the southeast (see, Fig. 1). Widespread use of pit latrines has been relevant for disposal of sewage in Tehran and Alborz provinces. Sewage contains high levels of nitrogen compounds (N-compounds) which can elevate the nitrate level in the aquifer^73^. Although Tehran Province Water & Wastewater Company has started to establish the required infrastructures for sewage collection and treatment, still some major regions of the city use the pit latrines for wastewater disposal. In addition, the unbalanced use of N-compounds’ fertilizers (annually around 200 kg/ha) for nourishing the green spaces and parks across the city introduces a large nutrient load to the aquifer^33,47,61^. The southeast part of the plain has mainly been covered by farmlands, which is an agricultural hub to meet the food demands for Tehran and Alborz provinces and nearby cities^45^. These farmlands are supported by unbalanced use of fertilizers^15,40^, which mainly include N-compounds (e.g., urea, ammonium sulfate, ammonium nitrate, and calcium nitrate), P-compounds (e.g., mono-ammonium phosphate and mono-potassium phosphate), K-compounds (e.g., potassium nitrate, potassium chloride, and potassium sulfate), and animal waste-based fertilizers such as cattle and poultry manures. These fertilizers can penetrate down through the farmlands and increase nitrate concentration in the aquifer. In addition, north to south direction of the slope in Tehran city brings nutrient rich-runoff during the rainfall and drainage of the pit latrines to the south, leading to further deterioration of water quality with nitrate in the aquifer^74^.

**Figure 7:**
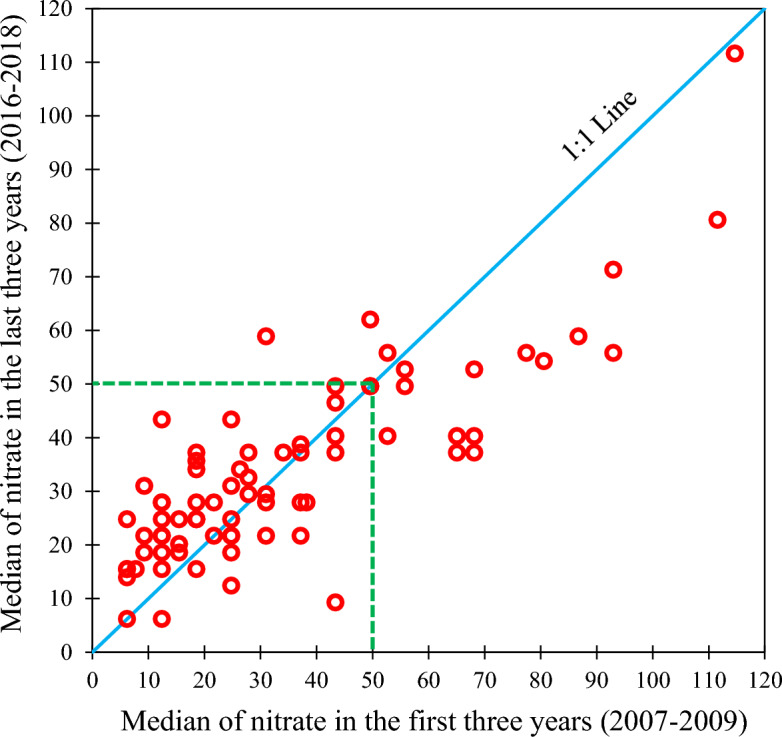
Comparison of the median nitrate concentrations in 75 wells sampled in the first three years and those that measured during last three years of the study period The dash green line determines the permissible level of nitrate for drinking water, i.e. 50 mg/L, as suggested by World Health Organization (WHO)^37^. This figure was prepared in Microsoft Excel.

The progress of the sewage collection network and construction of wastewater treatment plants during the last two decades is expected to reduce the input of nutrient loads to the aquifer. But our trend analysis results showed no significant change in nitrate concentration during the study period. Based on the results, nitrate concentration fluctuated in the aquifer from 2007 to 2018. The increase in the population of Tehran and Alborz provinces, as well as the intensification of both agricultural and industrial activities, which all contribute to the production of more sewage and input of nutrients to the aquifer, seems to neutralize the positive impact of progress in infrastructures on the nitrate of the groundwater resources in this plain. To further understand whether the nitrate has raised or declined in the aquifer, we plotted the median nitrate concentrations in 75 wells sampled in the first three years (i.e., 2007–2009) against those sampled in the last three years (i.e., 2016–2018) of the study period (Fig. 7). In this figure, the 1:1 blue line represents where the values would plot in the case there were no change between the first and last three years. As shown in Fig. 7, the median concentrations of nitrate in approximately 54% of the wells has decreased in the last three years of the study period. Also, the last three years showed high concentrations of nitrate (> 50 mg/L) compared with the beginning of the study period. Therefore, it could be concluded that increase in coverage of sanitation infrastructures has improved the groundwater quality by reduction of nitrate concentration in the Tehran–Karaj aquifer. The decline in recharges may also have a positive impact on the reduction of nitrate concentration because it could hinder the infiltration of nutrient-rich effluents into the aquifer^75^.

Water pollution by nitrate is considered as a global concern with profound implications for human health. Naturally, pristine groundwater resources contain a low level of nitrate, and it is supposed that aquifers with a nitrate concentration higher than 10 mg/L are likely exposed to anthropogenic sources of nitrate pollution^76^. Many regions throughout the globe have shown high nitrate levels in their drinking water. In a study conducted in a small region of the southern Tehran–Karaj aquifer^44^, 23 out of 148 taken water samples (~ 16%) revealed nitrate levels exceeded 50 mg/L. Also, Ghahremanzadeh et al.^40^ reported high levels of nitrate in the southern parts of Tehran–Karaj aquifer. These studies further confirm our findings about the concern on high levels of nitrate in some regions of Tehran–Karaj aquifer. Using the groundwater samples taken in west of Tehran city in 2016, Shirazi et al^77^ reported no concern about the elevated concentration of nitrate in this region—a finding that is in-line with our results as shown in Fig. 4. In another study conducted by Zendehbad et al.^78^, around 42% of the groundwater wells in the Mashhad region, northeast of Iran, showed nitrate levels above 50 mg/L, which is more than two-times greater than that in our study. However, more elevated nitrate concentrations than the maximum level observed in our study (i.e., 148.8 mg/L in 2010) have been reported in groundwater resources around the world. For example, Mukherjee and Singh^28^, concluded that the nitrate levels in Lower Ganga Basin, India, varied from 0 to 508 mg/L and 0 to 435 mg/L during the pre-monsoon and post-monsoon periods, respectively, in 2015–2016. Xu et al.^79^ concluded that human activities led to high concentrations of nitrate in groundwater of Guanzhong Basin, China, up to 397 mg/L, which raised the calculated HQ for some samples up to 14. As reported by El Amri et al.^80^, nitrate level in Mahdia-Kssour Essef aquifer, Tunisia, varied from 17 to 521 mg/L during 1998–2017. Muhib et al.^81^ reported that nitrate levels in groundwater resources in Bangladesh reached up to 253 mg/L, which exceeded the WHO^37^ and the other guidelines.

## Conclusions

Clean-groundwater resources support the life on our planet and protect the health of human who uses aquifers as the main source of water across the world. Using a rich dataset measured seasonally/annually in more than 75 wells from 2007 to 2018, we evaluated the nitrate concentration in the Tehran–Karaj aquifer as the most important supply of water needs for a population of more than 18 million. Also, we mapped the non-carcinogenic risks of nitrate in the aquifer to highlight the negative impacts of this pollutant in drinking water for health risk of different end-users. Our findings revealed some concerns regarding the high concentration of nitrate and its associated health risks in drinking water in the eastern and southeastern regions of the aquifer, where is densely populated by the residents of Tehran’s city and farmlands. Further investigations showed that nitrate has somewhat decreased during the study period, likely due to the progress of the sewage collection network, construction of wastewater treatment plants, and the declining trend of groundwater recharge due to frequent droughts and improved sanitary infrastructures. Our study’s findings suggest still further efforts are required to reduce the nitrate concentration below the permissible level for drinking water, i.e., 50 mg/L, across the aquifer. This is doable through complete coverage of the sewage collection network, sustainable agricultural practices, and balance use of fertilizers in farmlands, greenspaces and parks in the plain. Understanding how nitrate will change in the aquifer and what drives these changes under the impact of both climate change and human-man activities would help policymakers to protect the groundwater quality in this densely populated zone—a main task that needs further studies.

## Data Availability

The raw data used in this study are included in a “Zenodo” repository, available through https://zenodo.org/deposit/8179225.
